# Scientific Items

**Published:** 1880-01-20

**Authors:** 


### SCIENTIFIC ITEMS.

**Dangers of the Telephone.**—The introduction of new inventions amongst the practical requirements of civilized life brings with it its disadvantages. The telephone is destined to become a most useful agent in daily intercourse, but Dr. F. M. Peirce, of Manchester, points out, in British Medical Journal, a possible source of inconvenience in its use. The following case which came under his notice exhibits a way in which the ear may be more or less injured during the use of the telephone.

A woman about thirty-five years of age, manageress at a smallware manufactory in Manchester, which was connected with its office (two miles off) by a telephone, was listening to a message, when a violent clap of thunder took place, and which appeared to be conveyed through the wire. The effect on the listening ear was that of complete numbness and deafness, accompanied by a sensation of giddiness, slight nausea, and tinnitus aurium. These symptoms, with the exception of the deafness, passed away in a few minutes. Dr. P. did not see the patient for three or four days after this occurrence, and cannot, of course, speak as to the amount of deafness at first produced; but on the fourth day he examined the left ear (the listening ear), and found the hearing distance twenty forty-eighths of an inch. As his patient had always had perfect hearing with both ears and had never experienced any difficulty in hearing before, he thinks it very unlikely that this degree of deafness was due to any previous affection of the ear. She stated that she had never had anything the matter with her hearing until using the telephone during the storm. He had examined her lately, and found both ears and hearing distance quite normal; nearly a fortnight elapsed, however, before perfect hearing returned. This case was no doubt due to a concussion of the auditory nerve.—*Druggists' Circular.*

**Night Temperature at Different Altitudes.**—It is often warmer upon mountains than in valleys, especially in severe winters. Mr. Alluard states, in the *Comptes Rendus*, that he has found evidences at the observatory of Puy-de-Dome, of frequent similar inversions at night, by tracing:

1. The curves of minimum temperature, in the two stations of the observatory.
2. The curves of maximum temperatures, obtained under the same circumstances.
3. The curves of the mean temperatures of the two stations, deduced from the maxima and minima.

The curves of minimum temperature often intersect, in summer as well as in winter, so that the summit of the mountain is sometimes 5° (9° F.) warmer during the night than the base. There is no similar intersection in the curves of maximum temperature. The inversions occur most often when the upper and lower currents are from different quarters, but sometimes when the two currents have the same direction. Further observations are needed in order to find the cause of the anomaly.

**A New Philosophical Toy.**—A curious phenomenon of sound is the *singing book*, now a philosophical toy. Thanks to M. Pollard, naval engineer of Cherbourg, it is within every intelligent person's reach. You place a small book on a table, the floor, or a chimney piece, and presently it distinctly emits songs, or duets by a piano or harp, and violin solos. The book is composed of ordinary paper, leaves of the latter alternating with some of tin. The metal leaves are united, the last two with an electric current, thus forming a condenser. The top and bottom sides of the volume communicate with an electric wire, along the wall, but concealed, and terminating in a pile in another room, where the speaker or singer, etc., deposits the sounds of his voice in a wooden mouthpiece, containing a metal plate and a stylus which, touching a spring, sets free the electric current and transmits the sound to the book, where it is repeated—a phenomenon not yet capable of being satisfactorily explained.—*Kansas City Review.*

**A New Use for Electricity.**—A new and useful application of electricity has been made by an American inventor to the apparatus for reeling silk from the cocoon. The delicate filaments of silk are carried over wire arms, which are so nicely balanced that they do not press against the silk strongly enough to break it, and, in this relation a current is kept open; but if the filament breaks the arm falls, the circuit is closed, and an electro-magnet instantly stops the reel until the break is repaired. As the work is now done the detection of a broken filament depends entirely upon the skill of the workman, and the work must be carried on slowly that the eye can note any break, while with this automatic stop it is said the labor will be much more rapidly done and a more uniform thread produced. The invention is being introduced into France and Italy, the two great silk producing countries of Europe.

**Annual Deaths of the World.**—Has any one ever sought to know how many persons die annually throughout the world? First, we may cite some figures as to the total population of the earth, which may be stated at 309,000,000 for Europe, 824,000,000 for Asia, 199,000,000 for Africa, 4,500,000 for Oceanica, 85,000,000 for America, giving a total of 1,421,500,000 inhabitants of the world. Nearly 100,000 persons die annually in France, which gives 2,800 deaths per diem in round figures. But France is one of the most favored countries in a sanitary point of view. In many countries, where epidemics are almost continually prevailing, the mortality is one-third higher than in France. Still, taking the numbers of deaths as observed in France, we obtain as the total of the annual deaths for the whole world 35,693,350; *i. e.*, 97,790 persons die daily. As a compensation, the number of births is valued at 70 per minute, or 104,800 per diem.—*Union Med.; Medical Times and Gazette.*

**The Electrical Polyscope.**—A set of instruments called by this name were shown to the International Medical Congress, by M. Trouve, of Paris. He states that by them the interior of the stomach and bladder can be illuminated and examined with great completeness. Dr. Witsch, of Germany, has invented similar instruments, by which he claims to have attained the same results.—*N. Y. Med. Journal.*

